# Linkage to hepatitis C virus care in a binational cohort study of People who inject drugs on the U.S.-Mexico border

**DOI:** 10.1016/j.drugpo.2025.104899

**Published:** 2025-06-19

**Authors:** Bo Shan Go, Daniela Abramovitz, Irina Artamonova, Alicia Harvey-Vera, Natasha Martin, Gudelia Rangel, Christian B. Ramers, Samantha Tweeten, Winston Tilghman, Steffanie A. Strathdee, Robert G. Deiss

**Affiliations:** a University of Amsterdam, Amsterdam University Medical Centre, the Netherlands; b Division of Infectious Diseases and Global Public Health, Department of Medicine, University of California, San Diego, La Jolla, CA, United States; c US.-Mexico Border Health Commission, Tijuana, Baja California, Mexico; d El Colegio de la Frontera Norte, Tijuana, Baja California, Mexico; e Laura Rodriguez Research Institute, Family Health Centers of San Diego, San Diego, CA, United States; f Gilead Sciences, Foster City, CA, United States; g Epidemiology and Immunization Services Branch, Department of Public Health Services, County of San Diego Health & Human Services Agency. San Diego, CA, United States; h HIV, STD and Hepatitis Branch, Department of Public Health Services, County of San Diego Health & Human Services Agency. San Diego, CA, United States

**Keywords:** Hepatitis C virus, Substance abuse, Intravenous, Drug use, Opiate substitution treatment, HIV

## Abstract

**Background:**

People who inject drugs (PWID) experience high rates of Hepatitis C Virus (HCV) infection in the U. S.-Mexico border region, but their care continuum is poorly characterized.

**Methods:**

We analysed the HCV care continuum (linkage to care, initiation and completion of treatment) in a cohort of PWID in Tijuana, Mexico and San Diego. We also used multivariable Poisson regression to identify factors associated with linkage to HCV care among PWID in San Diego and Tijuana who reported prior HCV diagnosis.

**Results:**

Among 133 PWID with active HCV infection in San Diego, 50.4 %, 16 % and 14.3 % reported prior awareness of their diagnosis; linkage to care and treatment completion; corresponding proportions for 21 PWID in Tijuana were 19 %, 14.3 % and 0 %. In multivariable analysis, factors independently associated with increased linkage to HCV care included receiving medication for opioid use disorder (MOUD) (Adjusted Prevalence Ratio [adjPR] 1.38; 95 % CI: 1.23–1.54) and HIV-seropositivity (adjPR 1.87 (95 %CI: 1.32–2.66). Tijuana residency (adjPR: 0.73; 95 % CI: 0.70–0.75) and younger age (adjPR 0.94 per 5-year age decrease; 95 % CI: 0.89–0.99) were independently associated with decreased linkage to care. Among PWID with a prior HCV diagnosis, 52.6 % and 40.7 % reported linkage to care in San Diego and Tijuana respectively.

**Conclusions:**

Our study shows poor linkage to HCV-related care among PWID in both San Diego and Tijuana, along with low treatment completion. MOUD programs could be an efficient touchpoint to improve access to HCV care.

## Introduction

Approximately 2.2 million people are living with Hepatitis C virus (HCV) infection in the United States (US) (CDCMMWR, 2022), with injection drug use accounting for the majority of new cases (Trickey et al., 2019). Each year, 15,000 people in the U.S., 70 % of whom are people who inject drugs (PWID), die of complications from untreated HCV infection, making it the leading cause of liver-related mortality (Bell et al., 2008). Largely driven by the expanding opioid epidemic (Gomes et al., 2018), the number of new HCV infections in the U.S. increased almost six-fold between 2013–2022 (*2022 Hepatitis C* | *Viral Hepatitis Surveillance Report* | CDC, 2024a). While the number of HCV infections in the U.S. (67,400 cases in 2022) is decreasing, the rate of infection is still almost double the annual target (*2024 Viral Hepatitis National Progress Report* | CDC, 2024b) set by the World Health Organization (WHO) (Global Health Sector Strategies on HIV, Viral Hepatitis & Sexually Transmitted Infections for the Period 2022–2030, 2022) which aims to diagnose 90 % and treat 80 % of those infected with HCV while reducing HCV-related mortality by 65 % by 2030 (Global Health Sector Strategy on Viral Hepatitis 2016–2021. Towards Ending Viral Hepatitis, 2016). As such, the U.S. is not on track to meet the WHO’s HCV elimination goals.

By 2020, six years into the direct antiviral agent (DAA) era, only one-third of eligible, insured people living with HCV in the US were receiving treatment, with the number likely being lower for uninsured patients and PWID (Thompson, 2022). Traditional longstanding barriers to effective HCV care among PWID have included lack of knowledge about HCV and treatment options, internalized stigma (Batchelder et al., 2023; Crowley et al., 2018) and discriminatory attitudes and treatment restrictions (such as ongoing drug or alcohol use or HIV co-infection) (Asher et al., 2016; Barua et al., 2015; Hajarizadeh et al., 2018; Litwin et al., 2019). Despite increasing availability of treatment and the removal of these restrictions by Medicaid (Furukawa et al., 2024; Martin et al., 2023; Nephew et al., 2022), disparities in outcomes persist (Furukawa et al., 2024; Nephew et al., 2022). An additional factor underlying the low rate of linkage to HCV care is the widespread lack of integration of HCV care into harm reduction services which are frequented by PWID (Greenwald et al., 2023; Schwarz et al., 2022; van Santen et al., 2023). Achieving HCV elimination goals therefore depends on a comprehensive understanding and implementation of the HCV care continuum in a local setting that adapts to locally identified barriers, from screening to achievement of sustained virologic response (SVR).

Transnational migration and cross-border dynamics are known to influence the prevalence and character of substance use among Mexican immigrants in native and destination countries (Borges et al., 2007). Tijuana, Mexico and San Diego, California (heretofore San Diego) are located on a prominent drug trafficking route between Mexico and the US where large populations of PWID reside. In 2018, there were >55, 000 adults with a history of HCV infection in San Diego, one-third of whom were PWID (Wynn et al., 2021). This corresponds to a prevalence of 2.1 % among the general population and a 65 % prevalence among PWID; an extremely high burden of HCV compared to the US national estimate of 0.9 % (Lewis et al., 2023). In Tijuana, HCV seroprevalence among PWID was estimated at 35.2 % in 2020–21 (Marquez et al., 2024), compared to 95 % in 2005 (Frost et al., 2006). In the Mexico-U.S. border region, a recent study of PWID in San Diego and Tijuana found that the use of illicitly manufactured fentanyl was associated with higher rates of HCV seroconversion (Friedman et al., 2024). Although some progress in HCV treatment uptake was achieved in San Diego through a micro-elimination program specifically for people with HIV/HCV co-infection (Cheema et al., 2023), it is currently unclear whether San Diego and Tijuana are on track to reach HCV elimination goals.

Via cross-sectional analysis of a cohort of PWID in San Diego and Tijuana, we sought to characterize the HCV care continuum, focusing on barriers to and facilitators of linkage to HCV care in the region that could play a key role in strengthening the HCV care continuum for PWID. We also sought to identify touchpoints – particularly events where people could have been linked to HCV care – to explore opportunities to improve the overall continuum of HCV care. Last, we aimed to identify factors associated with linkage to HCV care among PWID in San Diego and Tijuana who reported prior HCV diagnosis, hypothesizing that PWID who are enrolled in syringe service programs (SSPs) or medication for opiate use disorder (MOUD) programs would be more likely to have been linked to HCV care.

## Methods

### Study design

Participants were selected from *La Frontera*, a cohort study of PWID aged 18 or older, who live in San Diego, California or Tijuana, Mexico, and who reported injection drug use in the last month (Strathdee et al., 2021). As described previously, participants were recruited in two waves: between October 2020–2021 and between February-June 2022 (Strathdee et al., 2021). These recruitment waves had slightly different inclusion criteria, as the original study aim was to research the relationship between border crossings and infectious disease transmission. Wave One thus consisted of three different recruitment groups: a) San Diego residents who crossed the U.S.-Mexico border for drug-related purposes (using or buying drugs) in the past two years; b) San Diego residents who did not cross the border, and c) Tijuana residents who did not cross the border. For Wave Two, only San Diego residents were included as required by the funder.

### Data collection

Participants were approached by trained, bilingual (English and Spanish speaking) study employees in areas with a known high density of PWID such as canyons, parks, abandoned lots and shelters (Bazzi et al., 2022). Following informed consent, each participant underwent HCV antibody testing and completed a bi-annual survey, receiving $20 per visit. Data collection was done either through outreach using a mobile van, in person at a storefront location, or on the phone or videoconferencing when COVID-related restrictions affected study operations. The study protocol was approved by the Institutional Review Board at Xochicalco University in Tijuana and the Human Research Protection Program of the University of California San Diego.

### Survey measures

Surveys were administered by trained bilingual interviewers using computer assisted personal interviewing (CAPI). The baseline survey consisted of questions covering demographic information (e.g., age, sex assigned at birth, ethnicity, education level, income and housing status), types of drugs used, frequency of drug use, injection practices (including whether or not someone made use of a hit doctor (i.e. someone who helps someone else inject drugs, usually in exchange for money or drugs) and sexual behaviour. With respect to HIV and HCV testing/treatment, we asked whether participants had ever been told by a doctor that they had tested positive for either. Those who answered affirmatively were asked whether they had been offered or initiated treatment after their last positive test, and whether they completed treatment (in the case of HCV infection). Regarding possible touchpoints where people could have been linked to HCV care, we asked whether participants were enrolled in a MOUD program ever or in the last six months and whether participants experienced incarceration or overdose (with or without medical attention) in the last six months. Completion of the survey took approximately 60 min.

### HCV testing

Participants underwent collection of venous blood samples and were screened for HCV through rapid antibody testing using Miriad© combined HIV/HCV antibody test in San Diego (MedMira Laboratories Inc., Halifax, Canada; sensitivity: 79 %– 88 % and specificity: 100 % (Tang et al., 2017)); in Tijuana, Accutrak^®^ HCV (sensitivity:100 %, specificity: 97–99 %) and Advanced Quality^®^ (sensitivity: 99 %, specificity: 99 %) were utilized. These point-of-care typically required 30 min, and results were provided to participants during the same baseline study visit. Samples testing HCV-seropositive subsequently underwent qualitative HCV ribonucleic acid (RNA) testing to confirm active HCV infection at the University of California San Diego School of Medicine, Centre for Advanced Laboratory Medicine. Study participants were contacted via telephone and notified of HCV RNA-positive results.

### Analytic samples

**Fig. 1.**
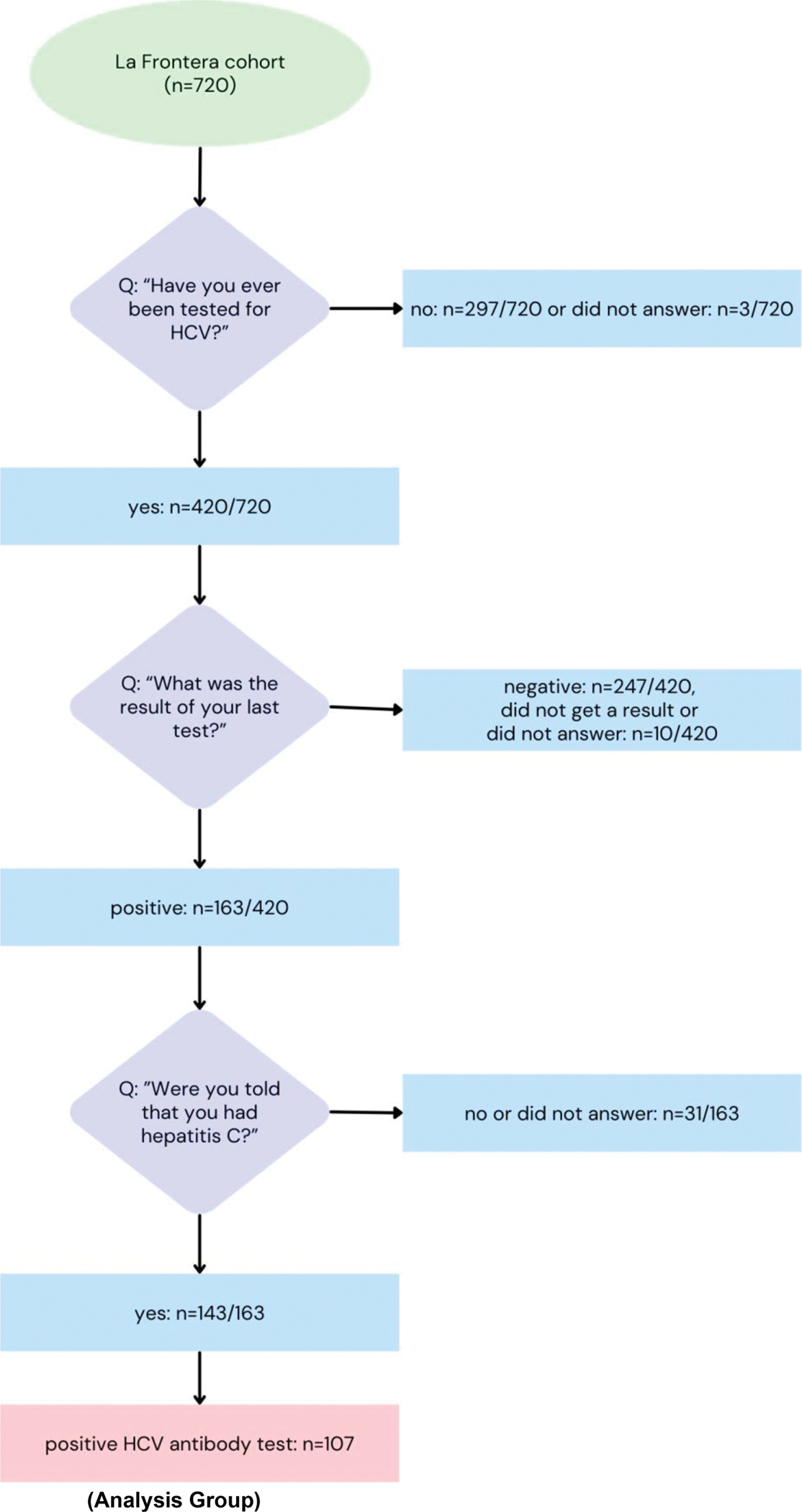
Study flow diagram. Flowchart demonstrating how the La Frontera cohort of PWID in San Diego and Tijuana (green circle) was used to select ‘Analysis Group’ for this study (red box). Blue boxes contain subsets of participants. Purple boxes contain questions from the survey.(Of the 720 participants, 612 were recruited in Wave 1, and 108 in Wave 2.)

Baseline survey data from all La Frontera participants (*n* = 720) were considered for this analysis. Individuals who tested HCV-antibody positive and reported having been told by a doctor at some point that they had HCV (*n* = 107) were considered to have been actively tested and diagnosed and to be eligible for treatment (Fig. 1). This group, considered at the time of the baseline interview to have been eligible for linkage to care, thus constituted our primary analytic sample (“Analysis Group”). Individuals from the full cohort who newly tested HCV RNA-positive but did not report a prior diagnosis (*n* = 83) were not included in this sample, as this group would not have had the opportunity to be linked to HCV care. All individuals receiving a diagnosis of active HCV were notified of test results and provided with referrals for HCV care.

**Fig. 2.**
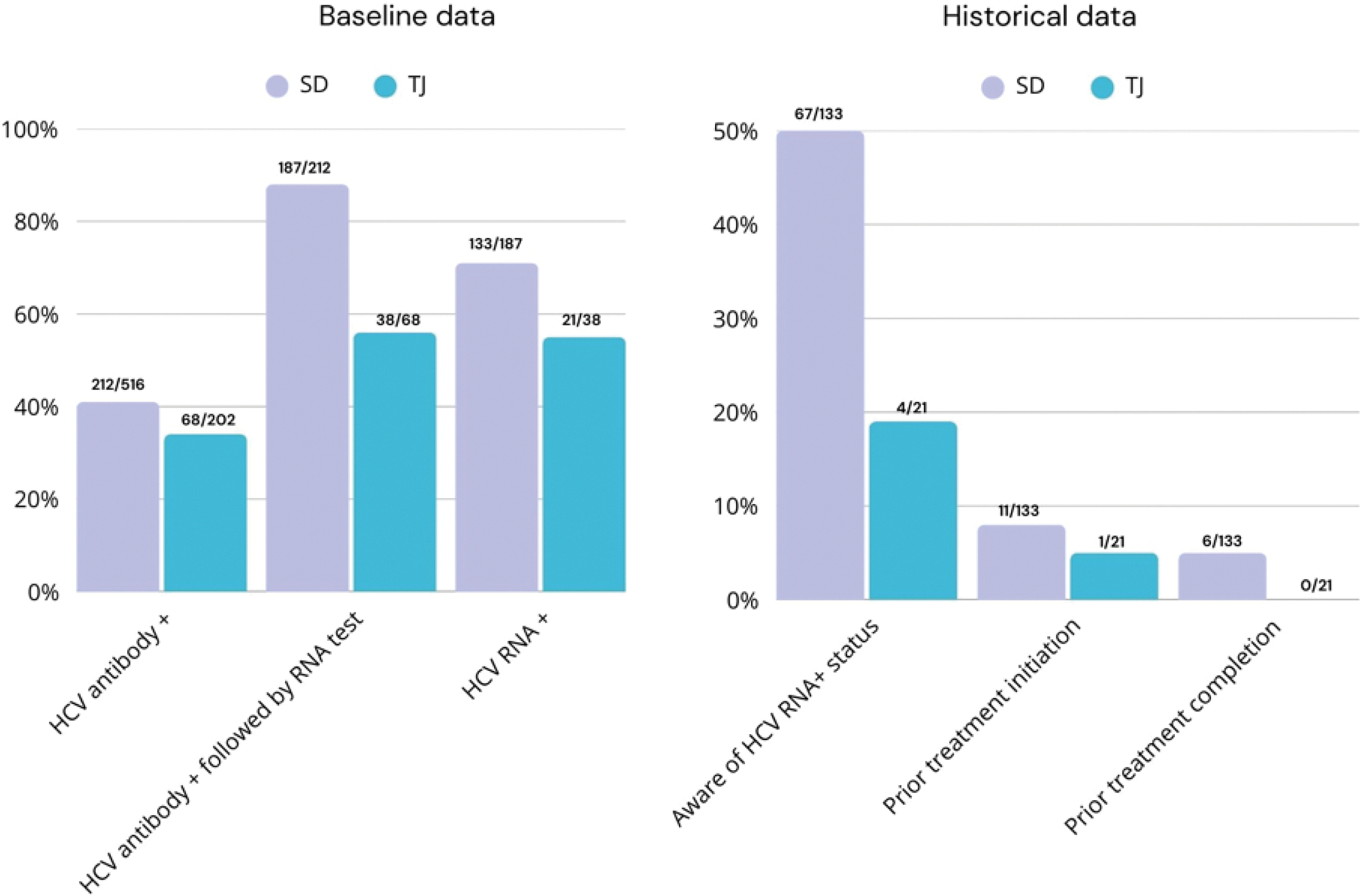
HCV care continuum among La Frontera participants in San Diego, CA and Tijuana, MX. Bar charts displaying percentages (with absolute numbers above each bar) of the number of PWID within the La Frontera cohort for different steps within the HCV care continuum. The left panel shows HCV testing data collected at baseline. The right panel shows retrospective, self-reported data which was collected through baseline visit questionnaires among individuals who tested HCV RNA-positive. SD = San Diego residents. TJ = Tijuana residents.

In addition to analysing the subset of participants who reported awareness of their HCV diagnosis in their baseline survey, we examined the historical continuum of HCV care for the full La Frontera cohort with active HCV (positive HCV antibody rapid test followed by a detectable HCV RNA test; *n* = 154) at their baseline visit (Fig. 2). We reviewed survey responses to determine how many individuals knew their HCV diagnosis, and how many had started and completed treatment. Individuals with active HCV infection who reported completion of treatment were presumed to have failed to achieve SVR or to have been re-infected. We attempted to verify self-reported treatment data by accessing medical records and cross-checking first/last names with major health centres and the health registry of County of San Diego’s Health and Human Services Agency. All participants for whom clinical data were requested provided a signed medical release at the time of study enrolment.

### Statistical analysis

Our definition for linkage to care was determined by answer to the question, “Has a doctor or health care worker ever offered you treatment for HCV?” as a discrete event between diagnosis and treatment initiation. Baseline characteristics were selected *a priori* and compared between the linked-to-care group and the not-linked-to-care group. We used either Chi-Square tests or Fisher’s exact tests for binary or categorical variables and Mann-Whitney U test for continuous variables to compare and describe the two groups. Characteristics of interest included demographic factors, drug use characteristics and potential touchpoints where participants could have been offered HCV treatment.

We built univariate and multivariable models to identify factors associated with being linked to care. Because a relatively high proportion of participants had been offered HCV care, we used Poisson regression to avoid overestimating relative risk. Univariate and multivariable Poisson regression with robust variance estimation via marginal generalized estimating equations (GEE) were used to estimate prevalence ratios along with corresponding 95 % confidence intervals. Furthermore, since participants were recruited at two different time points (Wave one and Wave two), at two different locations (San Diego and Tijuana), we controlled for potential correlations within recruitment groups by specifying recruitment group as a cluster effect with an exchangeable covariance structure.

For building the multivariable model, we used a “purposeful selection of variables” approach as first described by Hosmer, Lemeshow and May (Hosmer et al., 2008). All variables that yielded p-values ≤0.10 in univariate regressions were considered for inclusion in the multivariable model. After controlling for age and gender, only variables that yielded a 5 % significance level in the multivariable Poisson regression model were retained. The final model was checked for multicollinearity by examining values of the variance inflation factors and largest condition indexes. All possible two-by-two interactions between variables in the final model were assessed and ruled out. All analyses were conducted using SAS v9.4.

## Results

### Analytic sample characteristics

Table 1 shows baseline characteristics for individuals reporting prior diagnosis of HCV; *n* = 107), stratified by whether or not they were linked to care. In this group, 52.3 % (*n* = 56) reported having been linked to care and 47.7 % (*n* = 51) reported not having been linked to care. Median age was 40.0 years (interquartile range (IQR): 35.0–52.0); 75.7 % were male, 54.2 % identified as Hispanic/Latinx/Mexican, 85.0 % lived in San Diego as opposed to Tijuana, 21.5 % of individuals reported crossing the border to obtain drugs (San Diego residents only), and 3.7 % tested positive for HIV. Of those that had an HCV RNA test done (*n* = 84/107), 71.1 % tested positive. The median number of years that participants injected drugs was 19.0 (IQR: 12.0–27.0). Most participants used more than one drug with the most common drugs being methamphetamine (94.4 %), heroin (85.0 %), fentanyl (60.7 %) and cocaine (23.4 %). Over the six months prior to baseline, participants injected drugs at a median of 2.5 times per day (IQR: 1.0–4.0) and 57.9 % participated in receptive needle sharing.

Regarding touchpoints where participants could have entered into HCV care within the last six months, 17.8 % had been incarcerated, 26.2 % had been enrolled in a MOUD program and 29.0 % had experienced an overdose within the last six months.

### Continuum of HCV care for PWID in the La Frontera cohort

Within the full La Frontera cohort and regardless of prior HCV testing, 718 individuals received an HCV antibody test (Fig. 2). Of these, 41 % (*n* = 212/516) of San Diego residents and 33.7 % (*n* = 68/202) of Tijuana residents had a positive antibody test (*n* = 280/718; 39 % overall), while 80.4 % (*n* = 225/280) underwent subsequent HCV RNA testing using baseline blood samples. Of the latter group, 68.4 % (*n* = 154/225) tested HCV RNA-positive, including 71 % in SD (*n* = 133/187) and 55.3 % in Tijuana (*n* = 21/38). Among all individuals with active HCV infection at baseline, 46.1 % (*n* = 71/154) reported previously having been told that they had HCV, 7.8 % (*n* = 12/154) reported having started treatment and 3.9 % (*n* = 6/154) reported completion of treatment. By place of residence, 50.4 % (*n* = 67/133) of San Diego participants reported previously having been told they had HCV, 8.3 % (*n* = 11/133) reported having started treatment and 4.5 % (*n* = 6/133) reported completion of treatment. Conversely, 19 % (*n* = 4/21) of Tijuana residents reported having been told that they had HCV, 4.8 % (*n* = 1/21) reported having started treatment and 0 % (*n* = 0/21) reported treatment completion.

Using available medical records for 22 participants who reported initiation of HCV treatment, we verified sustained virologic response (SVR) among two San Diego participants. An additional nine individuals from San Diego initiated treatment but documentation of SVR was not available; for the final ten participants (nine from San Diego and one from Tijuana), we were unable to verify any treatment history.

### Factors associated with being linked to HCV care

Univariate Poisson regression models (Table 2) show that Tijuana residents were 29 % less likely to be linked to care compared to San Diego residents (prevalence ratio (PR): 0.71; 95 % confidence interval (CI): 0.70–0.72). Having a monthly income below $500 U.S. Dollars was also associated with a lower prevalence of being linked to care (PR: 0.62; 95 % CI: 0.55–0.71) as was being involved in sex work in the past six months (PR: 0.87, 95 % CI: 0.78–0.97).

San Diego residents who crossed the border for drug-related purposes were more likely to be linked to care compared to people who did not (PR: 1.24, 95 % CI: 1.12–1.38), as were people who had been incarcerated in the last six months (PR: 1.27, 95 % CI: 1.00–1.63). Additionally, a trend was noted for increased linkage to care among people who were HIV seropositive (PR: 1.48, 95 % CI: 0.94–2.35). Older age also was associated with increased prevalence of linkage to care (PR per 5-year increase: 1.08; 95 % CI: 1.04–1.12).

With respect to choice of drug, use of heroin and/or cocaine in the last six months was associated with a higher prevalence of being linked to care, while using fentanyl or methamphetamine were associated with a lower prevalence of being linked to HCV care (Table 2). Individuals reporting use of a hit doctor were also more likely to report being linked to HCV care (PR 1.24; 95 % CI 1.22–1.27). Each additional year of injection drug use yielded a small (1 % per year) increase in the prevalence of being linked to care (PR: 1.01; 95 % CI: 1.00–1.02), and each additional injection per day, on average, was associated with an 8 % decrease in prevalence of being linked to care (PR: 0.92; 95 % CI: 0.85–0.99). Individuals who were enrolled in a MOUD program within the last six months were almost 1.5 times more likely to be linked to care compared to those who were not (PR: 1.46; 95 % CI: 1.36–1.58).

### Factors independently associated with being linked to HCV care in multivariable analysis

Table 2 shows results of the final multivariable model. After controlling for age, gender and recruitment group, being enrolled in MOUD within the last six months (adjusted PR [adjPR]: 1.38; 95 % CI: 1.23–1.54) and HIV-seropositivity (adjPR 1.87 (95 % CI: 1.32–2.66) were both independently associated with increased linkage to HCV care. Living in Tijuana as opposed to living in San Diego (adjusted PR: 0.73; 95 % CI: 0.70–0.75) was independently associated with decreased linkage to HCV care.

## Discussion

In our cohort of PWID in San Diego and Tijuana who reported being diagnosed with hepatitis C infection, only half had been linked to HCV care. Additionally, among individuals with active HCV infection, fewer than half were aware of their diagnosis, and <10 % had initiated or completed treatment. Being enrolled in a MOUD program and having tested HIV-seropositive were independently associated with a higher prevalence of being linked to HCV care, which have important implications for integrating services to improve HCV treatment uptake among PWID, particularly among cross-border populations.

Co-location of HCV treatment along with services for substance use disorder (SUD) is an important strategy to improve outcomes for both, consistent with our finding of increased linkage to care among MOUD participants. A previous meta-analysis (Platt et al., 2017) reported that opioid substitution therapy as well as SSPs can reduce the risk of HCV transmission and acquisition between 50 %– 74 %; additionally, higher rates of treatment completion have been noted following clinical stabilization via treatment of SUD (Dimova et al., 2013). Nonetheless, despite MOUD programs being a significant touchpoint in efforts to eliminate HCV, fewer than 15 % of MOUD programs in the U.S. offer on-site HCV treatment (Bini et al., 2012; Jones et al., 2019) and more commonly, referrals for HCV testing occur outside of opioid treatment programs (Frimpong et al., 2014). In Mexico, closure of the country’s only methadone manufacturing plant for over a year interrupted MOUD treatment across the country and access to MOUD remains severely limited. Beyond preventing the acquisition of HCV and increasing viral suppression, our study shows that MOUD has the additional positive effect of linking PWID with HCV to care, a necessary first step toward the actual goal of HCV treatment initiation and completion.

In addition to MOUD, the provision of collocated harm reduction services for SUD and HCV treatment has potential to improve outcomes for PWID with polysubstance use or stimulant use disorders. Polysubstance use itself is a unique risk factor for HCV infection (Wagner et al., 2021), and our cohort reported high prevalence of both methamphetamine and opiate use. In a number of settings, on-site provision of HCV treatment at syringe service programs has been found to improve rates of treatment initiation and completion (Butsashvili et al., 2020; Midgard et al., 2021; Eckhardt et al., 2022). Additionally, offering medication-assisted treatment for treatment of methamphetamine use disorder may parallel the benefit seen with MOUD, as use of naltrexone-bupoprion and mirtazapine have both been shown to decrease methamphetamine use (Coffin et al., 2020; Trivedi et al., 2021). Evidence supporting the use of naltrexone-bupoprion was published following most of our baseline recruitment, and thus, future studies may examine the potential linkage between MAT for stimulant use disorders with HCV treatment services.

In addition to utilization of MOUD, we found that an HIV diagnosis was associated with greater likelihood of being linked to care for HCV treatment. This finding is somewhat intuitive, as people living with HIV (PLWH) are more likely to be receiving continuous care where they may be directly treated or more easily referred for HCV-related care. Consistent patient-provider relationships has been shown to improve care engagement among PWID (Heidari et al., 2023), as can the integration of related services within the same clinic. For instance, treatment for opioid dependence has been shown to lead to increased viral suppression among PLWH (Springer et al., 2012) and improved health outcomes (Chuah et al., 2017; Lee et al., 2024; Moyer et al., 2008; van Santen et al., 2021; Zaller et al., 2007). Such effects have led to official recommendations to integrate HIV and SUD treatment services in the 2022 IAS-USA Guidelines for the Treatment and Prevention of HIV (Gandhi et al., 2023). Conversely, the improved linkage we observed to HCV care among PLWH again underscores that HCV care provision remains insufficient for individuals who do not have HIV co-infection.

At present, HCV treatment for PWID in San Diego is available primarily at specialty (infectious disease) health clinics, but the treatment landscape is fragmented and can be complicated to navigate, particularly for PWID. In San Diego, HCV is a reportable disease and therefore cases are identified by the county health department, but universal treatment has not yet been realized. While previous barriers such as prior authorizations, treatment restrictions related to fibrosis score, substance use, specialty provider and patient co-pays have been removed, immediate test-and-treat in the context of street-based services is not a reality. From the time of initial evaluation with rapid HCV Ab testing, a delay of 1–2 weeks still exists due to the process of verifying HCV RNA status and obtaining prescription delivery. PWID with HCV infection in San Diego are mostly referred for treatment via passive means (e.g. flyer or clinic phone number) and may experience many barriers to receiving treatment on an individual level (such as competing interests or not having a working cell phone), as well as interpersonal levels (shame and stigma) and structural levels (e.g. inadequate transportation or facility hours) (Motavalli et al., 2021; Ross et al., 2015). A survey that was distributed by the Eliminate Hepatitis C San Diego County Initiative (County of San Diego Department of Health and Human Services, 2019) revealed that only 29 % of HCV care providers’ locations actually offer on-site HCV medication, causing a large proportion to be lost to care even after being linked to a clinic.

In response, a number of low-barrier treatment strategies further offer promise to improve HCV treatment outcomes outside of traditional clinic locations. Enhanced services, including on-site services at health centres that serve populations with difficulty accessing care, in lieu of study referrals (Coyle et al., 2015). The first point-of-care rapid HCV RNA test was approved by the FDA which should help identify the proportion of HCV-infected persons in need of antiviral treatment. Telehealth, combined with mobile care, has the potential to connect individuals in rural communities with specialist services which are traditionally difficult to access (Bianchet et al., 2024). In California, approximately 25 “street medicine” providers in California now operate to directly reach unhoused individuals (Valenzuela, 2025). Presently, a pilot study in San Diego is attempting to address this problem by simplifying the HCV care model with fewer appointments and providing streetside treatment in the vicinity of a SSP (Klaman et al., 2024). Together, these low-barrier approaches hold great promise in improving the HCV care continuum.

Beyond challenges in San Diego, we found even lower linkage to care for care-eligible PWID living in Tijuana. Only 19 % of participants from Tijuana with active HCV were aware of their diagnosis, while very few individuals reported initiation of treatment, and none reported prior treatment completion. This is unsurprising given the high density of PWID in Tijuana who live in high-risk environments and face isolation, discrimination, high barriers to health care and limited access to harm reduction services (Borquez et al., 2018; Melo et al., 2018). In Tijuana, the overall HCV epidemic has been shown to be aggravated by compulsory drug abstinence programmes being the main type of drug rehabilitation available (Borquez et al., 2018; Marquez et al., 2020; White et al., 2007); such programs have been found to disrupt harm reduction services (Kamarulzaman et al., 2016), increase the frequency of syringe sharing (Cunningham et al., 2018) and increase HIV and HCV transmission (Dolan et al., 2016). Although HCV elimination targets have been shown to be feasible in Tijuana if harm reduction services were scaled up, the criminalization of drug use in Tijuana, which includes syringe confiscation by the police, continues to hamper HCV elimination efforts (Marquez et al., 2020; Saldana et al., 2022).

Among individuals with HCV infection, several sub-populations would benefit from targeted efforts and integration of HCV and SUD treatment services. We found that younger people were less likely to be linked to HCV care, highlighting the need for youth-specific services that reach PWID earlier in their lives. Before shifting to injection, individuals in the peri-injection initiation phase are typically younger (Ganapathi et al., 2021) and could be reached for testing and treatment via non-injection opioid dependence programs. In addition, reproductive health clinics represent promising venues for HCV-related care for people who have engaged in sex work. Such locations have been shown to be central in combating HIV and other infectious diseases, such as Mpox (Goldenberg et al., 2023; Karmarkar et al., 2024). Last, border crossings in our cohort were fairly common, with 25 % of individuals from San Diego reporting crossing the border to use drugs in Mexico in the last six months, and we interestingly found modest improvements in linkage to care among PWID who routinely cross the border. Providing services near the border targeted to individuals who cross the border regularly may also increase treatment uptake.

Incarceration serves as one final touchpoint for improving HCV treatment uptake, and not surprisingly, we found higher linkage to care rates among people who were recently incarcerated. This is consistent with evidence from multiple settings, demonstrating that incarceration is an effective moment to realize improved individual and community health outcomes (Morris et al., 2017). Johnson et al. (2023) recently reported high HCV testing and treatment uptake among inmates of several prisons in New England, and HCV treatment among incarcerated PWID has also been shown to reduce transmission within Australian correctional facilities (Hajarizadeh et al., 2023). Beyond the period of incarceration, Bromberg et al. (2023) found improved rates of MOUD referral and uptake among individuals under community supervision (e. g. probation) in Ukraine. Unfortunately, very few U.S. jails conduct routine HCV testing (Beckwith et al., 2015; Wurcel et al., 2021), and fewer than half offer MOUD (Flanagan Balawajder et al., 2024). Our findings suggest that integrating HCV care more widely into correctional facilities within California could have a positive impact on the HCV care continuum.

### Limitations

Our study had several limitations. First, our analysis was cross-sectional, which precludes causal inferences, and our use of stepwise regression illustrates the exploratory nature of our analysis. Our main outcome of linkage-to-care was defined as having been offered HCV treatment, which is potentially susceptible to recall and social desirability biases, and reasons for a potential discrepancy between treatment offer and initiation were not explored. Our finding of low treatment uptake is nonetheless supported by the percentage of individuals found to have active infection. Additionally, if individuals had overstated their medical care, we would be overestimating the number of people that are linked to HCV treatment, suggesting the real number might be even further away from WHO elimination goals. Given the fragmented landscape of HCV treatment, we were unable to verify treatment histories reported by most participants, the number of participants from Tijuana was notably small. Finally, our data on the continuum of care was retrospective among people with active HCV infection; prospective studies will be necessary to explore treatment outcomes in the group of individuals with active HCV infection. Future research should therefore focus not only on the earlier stages of the HCV care continuum (i.e. screening, diagnosis and linkage to care) but would ideally prospectively collect treatment data, including especially factors related to ongoing SVR.

## Conclusions

Despite a high prevalence of HCV infection among PWID in the San Diego and Tijuana border region, currently few were linked to care, and an even smaller number were successfully treated. MOUD programs could be an effective touchpoint to improve HCV disease burden in this population and should be expanded to arrange for integrated, on-site HCV treatment with fewer treatment barriers for PWID. Concerted efforts between social service providers, shelters and clinics to publicize these efforts could further expand HCV care and simplify the care continuum for its intended users. Together, these findings highlight the need for action and targeted intervention strategies if San Diego and Tijuana are to meet WHO Hepatitis C elimination goals.

## Figures and Tables

**Table 1:** Baseline Characteristics of HCV-care eligible PWID from the La Frontera cohort, stratified by self-reported linkage to HCV care.

Baseline Characteristics^a^	Linked to care *N* = 56	Not linked to care *N* = 51	Total*N* = 107	p^b^

***Sociodemographic Factors***				
Sex assigned at birth (male)	45(80.4 %)	36(70.6 %)	81(75.7 %)	.239
Median Age (IQR)	42.5(36.0,53.5)	38.0(35.0,49.0)	40.0(35.0,52.0)	.111
Hispanic/Latino/Mexican	33(58.9 %)	25(49.0 %)	58(54.2 %)	.304
San Diego Resident	50(89.3 %)	41(80.4 %)	91(85.0 %)	.198
Cross border drug use at recruitment	15(26.8 %)	8(15.7 %)	23(21.5 %)	.163
Married or Common law	10(17.9 %)	10(19.6 %)	20(18.7 %)	.817
Monthly income <500 USD	22(39.3 %)	33(64.7 %)	55(51.4 %)	.009
Unhoused*	36(64.3 %)	29(56.9 %)	65(60.7 %)	.432
Spent time in jail or prison*	12(21.4 %)	7(13.7 %)	19(17.8 %)	.298
***Substance Use Factors***				
Used heroin*	50(89.3 %)	41(80.4 %)	91(85.0 %)	.198
Smoked/snorted/inhaled/vaped heroin*	30(53.6 %)	14(27.5 %)	44(41.1 %)	.006
Injected heroin*	50(89.3 %)	41(80.4 %)	91(85.0 %)	.198
Used methamphetamine*	51(91.1 %)	50(98.0 %)	101(94.4 %)	.118
Smoked/snorted/inhaled/vaped methamphetamine*	43(76.8 %)	42(82.4 %)	85(79.4 %)	.477
Injected methamphetamine*	38(67.9 %)	41(80.4 %)	79(73.8 %)	.141
Used fentanyl*	31(55.4 %)	34(66.7 %)	65(60.7 %)	.231
Smoked/snorted/inhaled/vaped fentanyl*	26(46.4 %)	28(54.9 %)	54(50.5 %)	.381
Injected fentanyl*	25(44.6 %)	27(52.9 %)	52(48.6 %)	.391
Used cocaine*	17(30.4 %)	8(15.7 %)	25(23.4 %)	.073
Smoked/snorted/inhaled/vaped cocaine*	14(25.0 %)	6(11.8 %)	20(18.7 %)	.079
Injected cocaine*	10(17.9 %)	5 9.8 %)	15(14.0 %)	.231
Median # of years of injection drug use (IQR)	22.5(13.5,29.5)	18.0(11.0,26.0)	19.0(12.0,27.0)	.125
Median # of injections per day (IQR)	2.5(0.8, 3.3)	2.5(1.0, 4.0)	2.5(1.0, 4.0)	.087
Receptive needle sharing*	34(60.7 %)	28(54.9 %)	62(57.9 %)	.543
Distributive needle sharing*	34(60.7 %)	29(56.9 %)	63(58.9 %)	.686
Uses hit doctor*	17(30.4 %)	11(21.6 %)	28(26.2 %)	.302
Experienced Overdose*	16(28.6 %)	15(29.4 %)	31(29.0 %)	.924
Enrolled in MOUD*	19(33.9 %)	9(17.6 %)	28(26.2 %)	.056
Utilized SSP*	33(58.9 %)	30(58.8 %)	63(58.9 %)	.991
***Sexual Behaviors***				
Traded sex in exchange for money/goods/etc.…*	6(10.7 %)	7(13.7 %)	13(12.1 %)	.634
Male having sex with male (ever) ^Y2^	7(12.5 %)	10(20.4 %)	17(16.2 %)	.272
Has/had casual sex partner(s) ^Y1^	21(37.5 %)	20(40.0 %)	41(38.7 %)	.792
***HIV and HCV related factors***				
Tested positive for HIV	3(5.4 %)	1(2.0 %)	4(3.7 %)	.620
Positive RNA result at baseline ^Y14^	34(72.3 %)	36(78.3 %)	70(75.3 %)	.508
Indicated that they were willing to be treated for HCV, if they had HCV^Y2^	52(94.5 %)	46(92.0 %)	98(93.3 %)	.706
***Recruitment group***				.311
Wave 1: SD Resident Drug Tourist	13(23.2 %)	6(11.8 %)	19(17.8 %)	
Wave 1: SD Resident non-Drug Tourist	27(48.2 %)	24(47.1 %)	51(47.7 %)	
Wave 1: TJ Resident non-Drug Tourist	6(10.7 %)	10(19.6 %)	16(15.0 %)	
Wave 2: SD Resident non-Drug Tourist	10(17.9 %)	11(21.6 %)	21(19.6 %)	

a For the binary variables the affirmative category is presented.

* Past 6 months.

Y Missing values:

Y1 : *n* = 1.

Y2 : *n* = 2.

Y14 : *n* = 14.

b NOTE: Analytic methods used: For binary and categorical variables with >5 observations per cell, we used Chi-Squared tests, for binary variables with ≤5 observations per cell, we used Fisher’s exact tests and for continuous variables, we used Mann-Whitney tests.

**Table 2:** Associations between baseline characteristics and linkage to HCV care among HCV-care eligible PWID from the La Frontera cohort.

Baseline Characteristics	Univariate PR (95 % CI)^a^	Adj. PR (95 % CI)^b^	p-value

***Sociodemographic Factors***			
Sex assigned at birth (male vs. female)	1.32 (0.79,2.18)	1.29 (0.80,2.08)	.303
Age (per 5 year increase)	**1.08 (1.04,1.12)**	1.06 (1.01,1.12)	.016
Hispanic/Latinx/Mexican (vs. not	1.19 (0.82,1.73)		
Tijuana resident (vs. San Diego resident)	**0.71 (0.70,0.72)**	0.73 (0.70,0.75)	<0.001
Cross border drug use at recruitment (yes vs. no)	**1.24 (1.12,1.38)**		
Married or common law (yes vs. no)	0.93 (0.46,1.89)		
Monthly income<500 USD (yes vs. no)	**0.62 (0.55,0.71)**		
Unhoused(yes vs. no)*	1.17 (0.88,1.56)		
Spent time in jail or prison (yes vs. no)	**1.27 (1.00,1.63)**		
***Substance Use Factors***			
Used heroin(yes vs. no)*	**1.44 (1.14,1.80)**		
Smoked/snorted/inhaled/vaped heroin(yes vs. no)*	**1.68 (1.21,2.33)**		
Injected heroin(yes vs. no)*	**1.44 (1.14,1.80)**		
Used meth(yes vs. no)*	**0.61 (0.42,0.90)**		
Smoked/snorted/inhaled/vaped meth(yes vs. no)*	0.88 (0.50,1.53)		
Injected meth (yes vs. no)*	0.75 (0.42,1.36)		
Used fentanyl (yes vs. no)*	**0.81 (0.63,1.04)**		
Smoked/snorted/inhaled/vaped fentanyl(yes vs. no)*	0.86 (0.67,1.11)		
Injected fentanyl (yes vs. no)*	**0.87 (0.77,0.98)**		
Used cocaine(yes vs. no)*	**1.49 (1.29,1.73)**		
Smoked/snorted/inhaled/vaped cocaine(yes vs. no)*	**1.52 (1.21,1.93)**		
Injected cocaine(yes vs. no)*	**1.39 (1.20,1.60)**		
# of years of injection drug use (per one year increase)	**1.01 (1.00,1.02)**		
# of injections per day on average (per one additional injection)	**0.92 (0.85,0.99)**		
Receptive needle sharing (yes vs. no) *	1.12 (0.87,1.44)		
Distributive needle sharing (yes vs. no)*	1.08 (0.79,1.47)		
Uses hit doctor (yes vs. no)*	**1.24 (1.22,1.27)**		
Experienced overdose (yes vs. no)*)	1.00 (0.77,1.29)		
Enrolled in MOUD (yes vs. no)*	**1.46 (1.36,1.58)**	1.38 (1.23,1.54)	<0.001
Utilized SSP (yes vs. no)*)	1.02 (0.83,1.25)		
***Sexual Behaviors***			
Traded sex in exchange for money/goods/etc.…(yes vs. no)*	**0.87 (0.78,0.97)**		
Male having sex with male (ever) (yes vs. no) ^Y2^	0.74 (0.51,1.09)		
Male having sex with male (yes vs. no)* ^Y1^	0.82 (0.35,1.91)		
Has had casual sex partners (yes vs. no)* ^Y1^	0.96 (0.77,1.19)		
Tested positive for HIV (yes vs. no)	**1.48 (0.94,2.35)**	1.87 (1.32,2.66)	<0.001

* Past 6 months.

Y Missing values:

Y1 : *n* = 1.

Y2 : *n* = 2.

ab Analytic methods used: aUnivariate and bmultivariable Poisson regression with robust variance estimation via Generalized Estimating Equations (GEE), controlling for clustering by recruitment group assuming an exchangeable covariance matrix (i.e., observations for any two participants belonging to the same recruitment group have equal correlations). Univariate prevalence ratios in bold yielded p-values ≤0.10.
